# Glycosylation of Cancer Extracellular Vesicles: Capture Strategies, Functional Roles and Potential Clinical Applications

**DOI:** 10.3390/cells10010109

**Published:** 2021-01-08

**Authors:** Álvaro M. Martins, Cátia C. Ramos, Daniela Freitas, Celso A. Reis

**Affiliations:** 1 Institute for Research and Innovation in Health (i3S), University of Porto, 4200-135 Porto, Portugal; ammartins@ipatimup.pt (Á.M.M.); cramos@ipatimup.pt (C.C.R.); 2 Institute of Molecular Pathology and Immunology (IPATIMUP), University of Porto, 4200-135 Porto, Portugal; 3 Instituto de Ciências Biomédicas Abel Salazar (ICBAS), University of Porto, 4050-313 Porto, Portugal; 4 Department of Chemistry, University of Aveiro, Campus Universitário de Santiago, 3810-193 Aveiro, Portugal; 5 Faculty of Medicine of the University of Porto (FMUP), 4200-319 Porto, Portugal

**Keywords:** extracellular vesicles, glycosylation, cancer, biomarker, therapy, detection, capture, exosomes

## Abstract

Glycans are major constituents of extracellular vesicles (EVs). Alterations in the glycosylation pathway are a common feature of cancer cells, which gives rise to de novo or increased synthesis of particular glycans. Therefore, glycans and glycoproteins have been widely used in the clinic as both stratification and prognosis cancer biomarkers. Interestingly, several of the known tumor-associated glycans have already been identified in cancer EVs, highlighting EV glycosylation as a potential source of circulating cancer biomarkers. These particles are crucial vehicles of cell–cell communication, being able to transfer molecular information and to modulate the recipient cell behavior. The presence of particular glycoconjugates has been described to be important for EV protein sorting, uptake and organ-tropism. Furthermore, specific EV glycans or glycoproteins have been described to be able to distinguish tumor EVs from benign EVs. In this review, the application of EV glycosylation in the development of novel EV detection and capture methodologies is discussed. In addition, we highlight the potential of EV glycosylation in the clinical setting for both cancer biomarker discovery and EV therapeutic delivery strategies.

## 1. Introduction

Extracellular vesicles (EVs) are small nano-sized particles, secreted by all cell types and capable of encapsulating and transporting several molecules to a target delivery site [1]. EVs can be found in various biological fluids and can be harvested in relatively non-invasive ways. Therefore, these particles are attractive systems for targeted drug delivery approaches and valuable sources of circulating cancer biomarkers.

**Figure 1:**
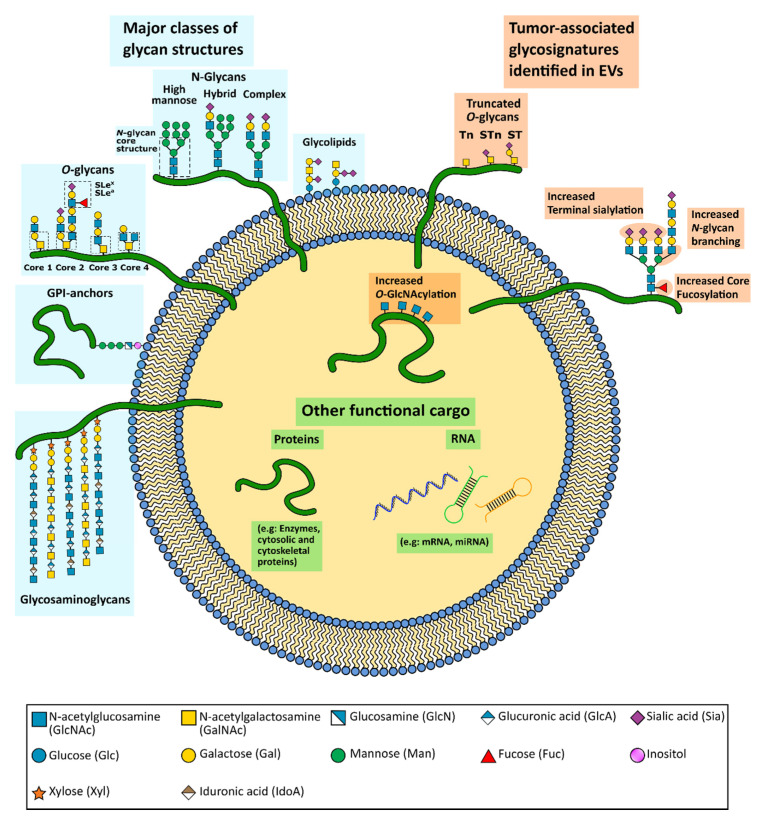
Schematic representation of an extracellular vesicle (EV) and its functional cargo. EVs carry a wide variety of functional molecules, including glycoproteins and glycolipids. The major common classes of glycoconjugates found in human cells are depicted on the left. Aberrant tumor-associated glycosylation already identified in cancer EVs are depicted on the right. The glycostructures were represented at the expected EV localization (intern or at the EV membrane) considering the knowledge from the cell glycans. Nevertheless, the specific localization of some of these structures is not yet fully elucidated.

Alterations of the glycosylation pathway are a common feature of malignant cell transformation [2,3,4]. These carbohydrates are capable of modulating several processes during cancer progression, including activation of oncogenic signaling pathways, interference with cell–cell and cell–extracellular matrix (ECM) adhesion and mediate cancer cell metastasis [3,4]. In addition, alteration in the glycosylation pattern of a cell has also been associated with content sorting processes [5,6,7], and with the capacity of cells to interact and uptake certain EVs [8,9,10,11]. Interestingly, some of the tumor-associated glycan alterations have already been identified as enriched in cancer EVs (Figure 1), which may constitute important biomarkers with the potential to be used in the clinical setting.

Despite technological advances, the structural characterization of glycans remains quite challenging. The diversity and complexity of these carbohydrates, together with methodological limitations, makes it challenging to deeply analyze the EV glycome [12,13]. The presence of specific glycosylation profiles in tumor EVs highlights its potential to be used not only to develop novel cancer EV detection and isolation methods but also as a source of novel circulating biomarkers. In this review, after a brief description of the main types of changes in glycosylation found in cancer and their impact on different pathological processes, we pointed out the challenges faced by the currently available methods used in the analysis of the EVs glycome. In addition, we summarized the glycosylation patterns already identified in tumor-EVs and discussed their known function in cancer and how they have been used to develop additional EV detection and isolation technologies. Finally, we highlight the potential clinical application of the EV glycosylation in anti-cancer therapy and in the discovery of new biomarkers for the prognosis and early diagnosis of different types of cancer.

## 2. The Impact of Glycosylation in Cancer Progression

Glycans are carbohydrate structures that modify both proteins and lipids through a biosynthetic pathway finely regulated by glycosyltransferases and sugar transporters. Glycans can be mainly found at the surface of the cellular membrane forming the commonly known glycocalyx, and they are essential mediators of cell–cell communication and cell–matrix interaction [3,14,15]. The major types of glycosylation that can be affected in cancer include (I) *N*-glycans, characterized by an *N*-linkage to an Asn residue in an Asn-X-Ser/Thr sequon, where X can be any amino acid except proline. *N*-glycans have a defined core structure, and can be classified depending on their structures and branches in high-mannose, complex or hybrid [16]; (II) *O*-GalNAc glycans, also known as mucin-type *O*-glycans, are carbohydrate chains initiated by a GalNAc sugar covalently linked by an oxygen atom to a Ser/Thr residue. They often appear as long ramified structures, and have multiple core structures, the major includes core-1 to core-4 [17]; and (III) glycosaminoglycans (GAGs), long and non-ramified carbohydrate chains consisting of repeating disaccharide units [18]. The glycosylation pathway is highly regulated by numerous players, including the expression, localization and activity of both glycosyltransferases and glycosidases, and the availability of nucleotide sugar donors [19]. Therefore, variations in the expression levels of specific glycosyltransferases [20] or its mislocalization in the endoplasmic reticulum (ER) and Golgi apparatus (GA) [21], dysregulation of chaperone activity [22] or alterations in nucleotide sugar transporter availability and cofactors [19] will result in the synthesis of aberrant glycosylation in cancer.

These macromolecules play pivotal roles in several physio- and pathological processes either by functioning as structural scaffolds, recognition cues or modulators of other important biomolecules [14]. Genetic and epigenetic alterations that disturb the glycosylation machinery often arise during malignant transformation, which results in loss or increased expression of certain glycans and the appearance of novel glycans [2,3]. The presence of aberrant glycosylation in cancer cells can impact several biological processes including tumor cell proliferation [23], angiogenesis [24], invasion [25] and metastasis [26]. Alterations in the glycosylation pathway provide multiple adaptive advantages, including receptor tyrosine kinase (RTK) activation [25,27,28,29], regulation of adhesion-related proteins [30,31,32] and immune response modulation [15,33], which significantly contribute to cancer progression. All these aspects will be briefly discussed, as they have already been extensively reviewed [3,4,34].

### 2.1. Receptor Tyrosine Kinase Activation

RTKs are a family of receptors that control several cellular signaling pathways involved in numerous biological processes, such as cell proliferation, growth, motility and differentiation [35]. Dysregulation of RTK activity is highly associated with cancer, which will impact the progression of the disease [36,37]. RTKs carry several glycosylation sites, and their hyperactivated status in malignant conditions is often associated with aberrant glycosylation. Namely, the increase in tumor-associated *N*-glycan branched structures promotes activation of different RTKs associated with cancer progression, such as the epidermal growth factor receptor (EGFR) [38,39,40], human epidermal growth factor receptor 2 (HER2) [41,42,43] and vascular endothelial growth factor receptor (VEGFR) [44,45]. The presence of aberrant glycans in cancer cells can directly regulate RTK–ligand binding and downstream signaling, and also stimulate receptor oligomerization through galectin-receptor binding, which increases the permanence of the receptor at the cell membrane [46]. Additionally, heparan sulfate (HS) chains can form cell membrane docking sites for ligands, including the hepatocyte growth factor (HGF) [47] and the vascular endothelial growth factor (VEGF) [47,48], resulting in enhanced binding and activation of MET and VEGF receptors, respectively.

### 2.2. Cell Adhesion Molecules

Integrins and cadherins are important cell receptor proteins responsible for the interactions between cells and the ECM [49]. However, their function can also be affected in cancer due to the presence of aberrant glycosylation, which consequently disturbs cell–cell and cell–ECM interactions [31]. These perturbations can be mechanically induced, when the glycocalyx of cancer cells promotes integrin clustering, thus increasing integrin signaling and adhesion [50]. Additionally, aberrant glycosylation can modulate the kinetic activity of this type of proteins, for example, the increase of α2,6 sialylation in breast cancer cell lines reduces the affinity of α5β1 and α2β1 integrins towards collagen IV and fibronectin [51]. Furthermore, overexpression of sialyl-Tn (STn) hinders the downstream signaling of α5β1 integrin causing significant morphological alterations in the cell [52]. Regarding the cadherins, *N*-glycan branching structures have been described to destabilize E-cadherin at the cell surface, and thus promote cell proliferation and tumor progression [53].

Selectins are another important type of cell adhesion molecule. These include E-selectin, P-selectin and L-selectin which are expressed in the vascular endothelium, platelets and leukocytes, respectively [54]. These lectins bind to terminal sialofucosylated structures, for example, sialyl-Lewis^x^ (SLe^x^) and sialyl-Lewis^a^ (SLe^a^), present in either *N*-glycans, *O*-glycans or glycolipids. The interaction between selectins and these terminal structures is crucial to the leukocyte “rolling” and extravasation process during inflammation [55]. By expressing SLe^x^ and SLe^a^, cancer cells are able to hijack this mechanism to interact with the selectins present in the vascular endothelium, traveling through the circulatory system and thus, potentiate their metastatic behavior [56,57].

### 2.3. Immune Response Regulation

Furthermore, glycans are crucial regulators of the immune response. Aberrant glycosylation can modulate the interaction between tumor cells and selectins, galectins or sialic acid-binding immunoglobulin-type lectins (Siglecs), which are the lectins involved in the immune response (reviewed in [15]). For instance, siglec-7 and siglec-9 are two inhibitor receptors expressed in natural killer (NK) cells that recognize and bind to sialic acids [58]. As a consequence, the increased sialylation in cancer cells induces inhibitory responses, hampering the ability of NK cells to effectively target and kill cancer cells [58,59,60]. Galectins are another important type of lectin in the immune regulation process, whose ligands are β-galactoside sugar residues. Interestingly, it was described that Galectin-9 can interact with Dectin-1, a C-type lectin, present in macrophages and program them into a tolerogenic state in pancreatic ductal carcinoma [61]. Additionally, galectin-3 has been reported to suppress CD8^+^ T cell responses towards tumor cells and causing T-cell anergy. This effect could be reverted through the depletion of galectin [62,63]. An additional example of relevant lectins of the immune system is the dendritic cell-specific intercellular adhesion molecule-3-grabbing non-integrin (DC-SIGN), which is present in macrophages. The interaction between this lectin and fucosylated glycans promotes tumor evasion from the immune response in breast cancer [64].

## 3. Shortcomings of Glycomic Approaches and Their Application in Extracellular Vesicle Glycome Analysis

In the past years, the field of glycomics has substantially evolved. Nevertheless, this evolution has been slower when compared to other-omics. The fact that glycosylation is not a template-based process, together with its complex structural diversity has slowed the development of tools and methods to accurately characterize glycans structurally [65,66]. Undoubtedly, the development of specific antibodies and plant-derived lectins for carbohydrate antigen detection has significantly boosted our ability to identify the presence of specific glycosylated structures. Lectins are proteins with binding specificity towards determined glycan structures, which can be immobilized in a microarray format and then be used to assess the overall glycome of a given sample [67]. Due to their high sensitivity, these lectins can potentially be used to probe the glycome of EVs from any source, such as cells or biofluids. Nevertheless, some lectins have overlapping binding affinities towards certain glycan structures. Therefore, these tools alone do not serve to provide a clean and comprehensive glycosylation profiling of a given sample. For a more extensive glycosylation analysis, advanced mass spectrometry techniques are required as they provide an in-depth glycosylation characterization of a given sample [68]. Indeed, the study of EV glycosylation has been rapidly growing in recent years. However, the yield and purity of current EV isolation approaches constitute limitations for the application of high-resolution EV glycomic methodologies [69]. Therefore, lectin-based approaches have generally been used to identify major glycan structures present in EVs from different sources [8,70,71,72,73], as we carefully detail in the following chapters. The majority of the studies approaching the EV glycome using mass spectrometry have been mainly focused on *N*-glycosylation. This is partially a consequence of the increased difficulty of decoding *O*-glycosylation [74]. To apply mass spectrometry methods for glycomic analysis, glycans must first be released from the sample, often through chemoenzymatic reactions. Although there is a specific enzymatic reaction to isolate *N*-glycans using PNGase F, there are no reliable enzymatic digestion protocols to isolate *O*-glycan chains [75,76]. It is possible to remove *O*-glycans using laborious chemical protocols, such as *β*-elimination. However, this chemical reaction may induce artifacts due to sample “peeling”, which is a phenomenon characterized by the isomerization and degradation of the innermost monosaccharides from the *O*-glycan chain [77]. Different conditions have been tested and successfully applied to reduce the “peeling” phenomenon, albeit not eliminating it completely [78]. In addition, *O*-glycosylation sites do not have a defined amino acid sequence, in contrast to *N*-glycosylation. Any Ser/Thr residue in a protein may or may not be modified with an *O*-glycan chain, making it more difficult to accurately predict *O*-glycosylation sites [17,79]. As a side remark, there are online resources, the NetOGlyc and the NetNGlyc, that predict putative *O*- and *N*-glycosylation sites, respectively, based on the provided protein/peptide sequence.

To overcome the challenge of *O*-glycosylation site disclosure, a genetic strategy targeting the *COSMC* gene, which codifies a key molecular chaperone that together with the C1GalT1 glycosyltransferase is responsible for the *O*-glycan elongation, was developed [80]. The *COSMC* gene knockout will lead to the shift of the *O*-glycosylation pathway towards the expression of simple forms of *O*-glycans, namely Tn and sialyl-Tn. The glycoengineered cell line was named “SimpleCell” (SC). By the targeted purification of the *O*-glycopeptides and further mass spectrometry characterization, this approach allowed the identification of the protein carriers of these structures and the disclosure of the respective *O*-glycosylation sites [80,81]. For instance, gastric SC models allowed the identification of novel important *O*-glycoproteins and their *O*-glycosites with biomarker potential for gastric cancer [82]. More importantly, this strategy has proved its usefulness in identifying novel biomarkers for cancer detection [83].

Different high-resolution quantitative mass spectrometry methods previously established to elucidate the *N*-glycome and *O*-glycome of cell lines have been adapted to the study of EV glycosylation. For example, the *N*-glycome of EVs isolated from glioma [71], melanoma [84] and ovarian carcinoma cell lines [72] has already been characterized. Additionally, the *N*-glycome of urinary EVs from prostate cancer patients has also been assessed using other high-resolution glycomic methods [85]. Regarding EV *O*-glycosylation, our group has demonstrated the presence of the tumor-associated antigen, STn by specific antibody recognition in gastric cancer EVs [86]. Other groups have also identified the presence of Tn and STn in cervical cancer cell line models using an innovative lectin-based approach [87]. Interestingly, also identified was the presence of proteins modified with *O*-GlcNAcylation, a type of intracellular *O*-glycosylation, in metastatic colorectal cancer EVs [88]. Outside of the cancer context, both the *N*- and *O*-glycosylation profile of EVs from healthy individual urinary samples was also disclosed using high-resolution glycomic techniques, such as matrix-assisted laser desorption ionization time-of-flight (MALDI-TOF) and liquid chromatography/tandem mass spectrometry (LC/MS-MS) [89].

Given the fundamental impact of aberrant cellular glycosylation in cancer progression and its association with patients’ poor-survival [3,4,25,90], we believe that a full characterization of cancer EV glycans by high-resolution approaches will disclose important cancer circulating biomarkers and set the ground to further explore its role in cancer biology.

## 4. Cancer Extracellular Vesicles Glycosylation

EVs are small nano-sized particles that are released into the extracellular space by all types of cells. These vesicles exert a broad array of biological functions, being important mediators of intercellular communication [1]. EV diameter typically ranges from 35–5000 nm, and therefore are quite smaller than cells, but much larger than proteins [91,92]. The general term EVs comprises three main types of vesicles which are classified, according to their size and biogenesis mechanism, into exosomes, microvesicles (ectosomes or microparticles) and apoptotic bodies. Exosomes have an endocytic origin and are produced by the inward budding of the plasma membrane of the cell. This invagination of the cell membrane leads to the formation of multivesicular bodies (MVBs) that can later either fuse with lysosomes for content degradation or fuse with the cellular membrane to be secreted in the form of exosomes. The formation and release of exosomes can be regulated by the endosomal sorting complex required for transport (ESCRT) [93,94]. In this type of vesicles, it is expected to find ESCRT proteins and accessory proteins for this complex, such as the ALG-2-interacting protein X (Alix), the tumor susceptibility gene 101 (TSG101) and the chaperones HSP70, Hsc70 and HSP90β, independently of the type of cell origin [92,95,96]. Another mechanism, independent of the ESCRT complex, can be used for exosome release [97]. In the absence of this complex, the endosome pathway may be regulated by the type II neutral sphingomyelase and the tetraspanin family proteins [98]. Therefore, exosomes will contain high levels of tetraspanins such as CD9, CD63 and CD81 [99]. On the other hand, microvesicles are released into the extracellular space by direct shedding of the plasma membrane [93,94]. Therefore, microvesicles can carry both cytosolic and plasma membrane proteins, including the same tetraspanins found on exosomes [100]. Microvesicles can also contain cytoskeletal and heat shock proteins and integrins [101,102]. Finally, apoptotic bodies are formed during the cellular apoptotic process. The process of cell apoptosis is characterized by the condensation of chromatin followed by the degradation of the internal structure of a cell [93,94]. The disintegrated cellular content will be part of the apoptotic bodies’ cargo. Therefore, this type of vesicles can contain proteins associated with several organelles such as histones (nucleus), the heat shock protein HSP60 (mitochondria) and the chaperone GRP78 (endoplasmic reticulum) [103,104,105].

EVs are composed of a phospholipid bilayer that provides protection to their cargo against degradation by the proteases and nucleases present in the external environment [106,107]. EVs encapsulate several molecules, including cytosolic and cytoskeletal proteins as well as enzymes and nucleic acids (mRNA, miRNA, tRNA, rRNA, DNA) [108,109,110]. The surface of the EVs is composed of lipids (ceramide, cholesterol, phosphatidylserine and sphingomyelin) and proteins (transmembrane proteins, antigen presenters and adhesion molecules) [1]. In addition, glycans are also relevant constituents of the EV composition surface [6,86,91,111].

Cancer EVs are able to mediate communication between cells locally and at a distance, and their cargo can influence the behavior of the recipient cell [112]. Importantly, tumor microenvironment stressors, such as hypoxia [113,114], acidosis [115], starvation [116,117], oxidative stress [118,119], radiation [114] and anti-cancer therapies [120], are important regulators of not only EV secretion and trafficking, but also of its molecular composition (as reviewed in [121]). EV cargo is mainly similar to the composition of the parental cell [1]. Although, they still have unique molecular profiles resultant from specific sorting mechanisms during the EV biogenesis process. Particularly, specific patterns of glycans were found enriched in EVs [5,6,122]. In cancer, important modifications occurring in surface glycans, both at cellular and EV level, may constitute important markers for EV detection, isolation and, importantly, for tumoral and non-tumoral EV distinction.

### 4.1. Extracellular Vesicle Surface Glycans as Relevant Markers for Its Detection and Isolation

The unique presence of certain EV surface glycans holds the potential to be targetable for the development of novel EV detection and isolation methodologies. Cancer EVs are rich in high mannose and complex type N-glycans, polylactosamine, as well as sialylated glycans, namely α2,6-linked sialic acids [6,123] (Figure 1). Detailed analysis of EV glycosylation can provide useful information to facilitate the distinction between different populations of EVs. So far, several strategies have been developed to characterize the glycan profile of EVs. Nevertheless, the identification of certain EV glycans can often fail due to technical difficulties. Importantly, an in situ rolling circle amplification strategy to enhance the detection capacity of the glycans present in cancer EV samples has been reported [87]. Another fluorescent-based strategy, the evanescent-field fluorescence-assisted (EFF) lectin array system, has also proved to successfully profile the glycans present in exosomes derived from mesenchymal stem cells [124]. These methods were able to quantify the detected glycans present in the EV samples due to the fluorescence emitted signals at the time of the glycan recognition. Therefore, these approaches hold the potential to distinguish the type and amount of glycans carried by EVs from different sources and/or to differentiate between the glycans present in EVs and their parent cells.

Different subpopulations of EVs may share size and biochemical characteristics, which makes it impossible to differentiate or specifically isolate them from a given sample [125,126]. In fact, most of the currently available methods for EV isolation will enrich different sub-populations of EVs carrying diverse molecular compositions [127,128,129,130]. Our group previously showed that different EV isolation techniques resulted in an enrichment of diverse EV glycosylated profiles, depending on the capacity of each method to separate cancer EVs from the non-EV content [86]. Furthermore, if EVs from in vitro cell cultures are used, culture conditions are critical for downstream EV glycosylation analysis [86]. Interestingly, asymmetric-flow field-flow fractionation (AF4) technology proved to efficiently isolate distinct EV sub-populations, including exomeres, carrying a specific glycan and protein profile, from a heterogeneous EV source [91]. Similar results were obtained when different EV populations were separated based on their tetraspanin profile and specific glycan signatures could be distinguished according to the host-cell type and each EV subpopulation [131].

Traditional isolation methods are based on EV size and buoyant density, namely, ultracentrifugation-based methods [132,133], microfiltration [134,135] and size exclusion chromatography [136,137]. Others explore the fact that EVs change their solubility and/or aggregate in different solutions, namely, by precipitation [137,138,139]. More recently, isolation methods were developed based on highly specific interactions with the exposed components on the EV surface, called immunoaffinity, or microfluidic technologies [140,141,142,143]. Nevertheless, there is no gold standard EV isolation method and each approach has its own advantages and disadvantages. As glycans comprise a major molecular component of EV surface, they constitute a valuable source of targets to capture and isolate EVs from a heterogeneous sample. Indeed, the use of the lectins peanut agglutinin (PNA) and *Artocarpus integrifolia* (AIA or Jacalin), that specifically bind to T-antigen, and the *Maackia amurensis* lectin I (MAL-I), that has a high affinity for Gal or GalNAc residues with an α(2,3)-linked sialic acid, allowed to isolate different sized urinary EVs from healthy samples based on their surface glycosylation profile, with increased yields and higher purity when compared to CD9/CD81/CD63 antibody-based isolation [144]. In addition, the STL lectin, recognizing the N-acetylglucosamine and lactosamine residues, also showed high affinity and specificity when isolating EVs from healthy urine samples [145]. One of the main challenges when using urine samples is the co-purification of the highly glycosylated Tamm–Horsfall protein (THP). This glycoprotein is able to form aggregates and capture EVs, hampering further EV biomarker downstream analysis [146]. However, the use of lectin microarrays allowed the distinction between EV glycosylation and THP glycosylation, which potentiated the isolation of urine EVs with minimal interference of this glycoprotein [70]. A similar approach was also applied to tumor EVs. By coupling a high mannose-type glycan-specific lectin to beads it was possible to capture small EVs from melanoma, glioblastoma, lung and colon cancer cells [147]. Interestingly, CD109, integrin α6 and ADAM10 present on melanoma small EVs were apparently responsible for the identified EV-lectin interaction, as they carry high mannose glycans [147]. These results demonstrated the potential of lectin-conjugated beads to detect and isolate different sub-populations of EVs within a sample, based on its glycosylation profile. In addition, a nanoparticle-based time-resolved fluorescence immunoassay (NP-TRFIA) showed to be able to capture EVs from urine samples and cell supernatants based on the interaction with the tetraspanins and glycan antigens present at the EV surface [148]. This approach also provided a general EV surface glycan profiling, which revealed a differential expression pattern of tumor-associated proteins on more aggressive versus less aggressive prostate cancer cell line-derived EVs [148].

Apart from the affinity-based isolation methods, a commercially available precipitation kit, the ExoGAG, was also developed taking into consideration the glycosylation profile of EVs. This method precipitates EVs out of the solution due to the presence of negatively charged GAG at the EV surface [149]. Interestingly, ExoGAG has proved to effectively isolate EVs from liquid biopsy patient samples with higher yields and purity when compared with UC and allowed the identification of Annexin A2 as an EV marker associated with endometrial cancer staging and recurrence [149].

Despite the technological advances in the field, the study of EVs is still technically challenging. Particularly, innovative and less laborious detection and isolation methods are an urgent need to facilitate an in-depth study of the different EV cargos as a reliable source of biomarkers. The presence of specific patterns of glycans at the EV surface constitute valuable sources to potentiate the development of more sensitive and specific EV detection and isolation methodologies, with the potential to be translated into the clinical setting.

### 4.2. The Functional Roles of Extracellular Vesicle Glycosylation in Cancer

Up to date, little is still known regarding the role of EV glycosylation in cancer. Nevertheless, the role and impact of glycosylation in EV biodistribution, uptake and protein cargo sorting have already been proven (Table 1).

#### 4.2.1. EV Biodistribution and Uptake

It has been described that alterations at the EV terminal sialylation resulted in a re-direction of the EVs to particular organs in mouse models [150]. In this work, the authors radiolabeled and treated EVs isolated from mouse liver progenitor cells with neuraminidase, a glycosidase that cleaves terminal sialic acid residues, and observed an increased accumulation of neuraminidase treated EVs in the lungs and axillary lymph nodes when compared to the non-treated EVs [150]. As increased sialylation is a common feature of cancer cells, exploring and understanding the role of terminal sialic acids in EV organ tropism may open new avenues to block cancer metastasis. Interestingly, EV sialylation also seems to play a role during the EV uptake by recipient cells [8]. The glycosylation profile of EVs isolated from murine hepatic cell lines was characterized and further manipulated by different glycosidases. For most of the cell lines tested, desialylated EVs had higher uptake efficiency. In other cases, cleavage of EV *N*-glycans by PNGase F digestion proved to be more efficient during EV uptake than neuraminidase treatment [8]. In a cancer context, it was also demonstrated that removal of the terminal sialylation through neuraminidase treatment also led to a small increase in EV uptake by ovarian cancer cells, although non-significant [152]. It is possible that the decrease in negative charge at EV surface, after sialic acid removal, could facilitate the interaction between EVs and recipient cells, due to the exposure of other carbohydrate ligands at the EV surface that allows for specific lectins to bind and facilitate the EV uptake [152]. In addition, it is established that the proteoglycans present at the cell membrane play major roles during EV-uptake [157]. For instance, the glypican and syndecan, two known heparan sulfate proteoglycans (HSPGs) at the cell surface, mediate the uptake of EVs in glioblastoma cells [9]. Nevertheless, the HSPGs present at the cell membrane are important mediators of EV internalization, but not vital, since perturbation of HSPGs synthesis does not completely inhibit EV uptake, suggesting the existence of HSPG-independent internalization mechanisms [9]. Altogether, these results interestingly suggest that EV and cell glycosylation can impact EV uptake and, therefore, drive specific EV organ biodistribution tropism.

Considering that the presence of certain glycans in cancer cells can regulate the immune response against tumor cells [3], it is possible that cancer EV glycosylation can also affect the immune system. For example, macrophages expressing siglec-1 use this receptor to capture sialylated pathogens [158]. It has been shown in vivo that these macrophages also incorporate highly sialylated EVs, especially enriched in α2,3-sialylation. This effect could be attenuated in siglec-1-deficient mice or when treating EVs with neuraminidase [159]. Considering the high prevalence of α2,3-sialylation in cancer, we can speculate that the presence of this type of glycans in tumor-derived EVs can mediate a communication axis between immune and cancer cells. Although some of the studies mentioned above were performed using non-cancerous cells, the results provided new insights into the potential roles that EV glycosylation modifications might play in a cancer context.

#### 4.2.2. Protein Sorting

Proteins are sorted into EVs through a variety of mechanisms depending on a multitude of factors, such as the class of EV and the protein to be sorted [93,160]. Proteins destined for extracellular vesicle secretion are clustered at the site of EV assembly, prior to their formation. From these proteins, a large portion will carry important glycan modifications [73].

There has been growing evidence supporting the importance of glycosylation in regulating EV protein sorting. Changes in the glycosylation profile acquired during malignant transformation may interfere with EV protein sorting. It was suggested that the conservation of specific glycan structures, such as increased high mannose, polylactosamine, α-2,6 sialylation and complex *N*-linked glycans and loss of terminal blood group A and B antigens is due to sorting mechanisms of glycoproteins and glycolipids into the EVs [6]. Additionally, it was also demonstrated that the sorting of EWI-2 into the EVs is regulated by its glycosylation, more specifically by complex *N*-glycans [161]. Both inhibition and site-directed abrogation of these complex *N*-glycans in EWI-2 hindered its recruitment to the EVs without altering its whole cellular localization [161]. Recently, it was reported that altered expression of the glycosyltransferase FUT8, responsible for the addition of fucose residues to the *N*-glycan chains, altered the proteome of secreted EVs in prostate cancer [162]. This study suggests that the aberrant expression of fucosylation in prostate cancer cell models leads to a decrease of endosomal sorting proteins in the EVs, such as the ESCRT and other clathrin-mediated endocytosis components, which results in altered protein cargo profiles in the secreted EVs [162]. Furthermore, aberrant *O*-glycosylation has also been reported as capable of affecting the sorting of some proteins into EVs, including the CD44 protein [163]. This heavily *O*-glycosylated transmembrane protein has been previously observed to be expressed in EVs carrying truncated *O*-glycan chains, namely STn [86]. It was observed in colorectal cancer cell line models expressing truncated *O*-glycans that CD44 is increasingly secreted via EVs when compared to the EVs of their normally glycosylated counterparts [163]. Interestingly, the role of ST6Gal1, a glycosyltransferase that catalyzes the transfer of sialic acids to galactose residues present in *N*-glycans in an α2,6 position [164], in EV protein cargo sorting has been addressed. Knocking-out ST6Gal1 in the SW620 colorectal cancer cell line model contributed to an increased tumor cell adhesion and migration, through the decreased expression of KAI1 in EVs [165]. This protein, a member of the tetraspanin superfamily, is described to inhibit several relevant signaling pathways during metastasis of cancer cells [166]. On the other hand, the sorting of some proteins into EVs was not affected by its glycosylation status. For instance, Lipocalin 2 (LCN2), a highly conserved protein that is secreted in EVs, has one *N*-glycosylation site which was speculated to be important in the trafficking of LCN2 into the EV [167]. However, it has been verified that this was not the case, since when treating several cell lines with tunicamycin, an inhibitor of *N*-glycosylation, LCN2 was still being sorted into EVs [167]. Considering all the studies mentioned above, the role of glycosylation in EV cargo sorting may be dependent on the type of cells and/or the protein to be sorted. Nevertheless, deciphering the role of glycosylation in cancer EV sorting might elucidate novel mechanisms to target cancer progression. It is known that EVs have the ability to reprogram recipient cells and regulate physiological processes both in health and disease conditions, due to their ability to carry and transfer functional cargo [168,169].

#### 4.2.3. Cell Behavior Modulation

Glycans, being part of the EV functional cargo, have been described to play important roles in the cell reprogramming process. EVs derived from breast cancer were able to induce invasion of the recipient cancer cells due to the transfer of the extracellular matrix metalloproteinase inducer (EMMPRIN) [170]. This effect was observed to be dependent on the *N*-glycosylation status of the EMMPRIN, more precisely on the Asn 160 and Asn 268 glycosylation sites [170]. In another study, treating ovarian cancer cell lines with EVs enriched in CD82 inhibited their cell adhesion. However, when treating the same cell line with EVs enriched with CD82 lacking the *N*-glycosylation site at Asn 157, no inhibition of cell adhesion in the recipient cells was observed [171]. EVs may also transport active enzymes that could modulate the recipient phenotypes [168]. For instance, Zhang Q et al. reported that cancer EVs carrying the ST6Gal1 enzyme induced an increase in the total α2,6 sialylation of the recipient cell [172]. Taking into account that α2,6 sialylation is a major alteration associated with increased cancer aggressiveness [3], this study suggests a possible mechanism by which EVs can induce cell tumorigenicity.

Considering the critical roles glycans play both in health and cancer situations, further deepening our understanding of the functional roles of EV glycosylation in cancer will open avenues to develop new tools for cancer diagnosis and therapy.

### 4.3. The Potential Clinical Application of Cancer Extracellular Vesicles Glycosylation

EVs are capable of carrying several bioactive molecules and, depending on their surface composition, hold the potential to be used as natural vehicles for localized drug delivery [173,174]. In addition, as cancer EVs can be found in several biofluids [148] and their cargo partially reflects the content of parental cells [1], these vesicles are also considered promising sources of circulating cancer biomarkers.

#### 4.3.1. EV Glycosylation as Therapeutic Delivery Tools

EVs can be re-engineered to carry specific molecules, either by manipulating their parental cells or by direct functionalization of the EVs [175,176].

**Figure 2:**
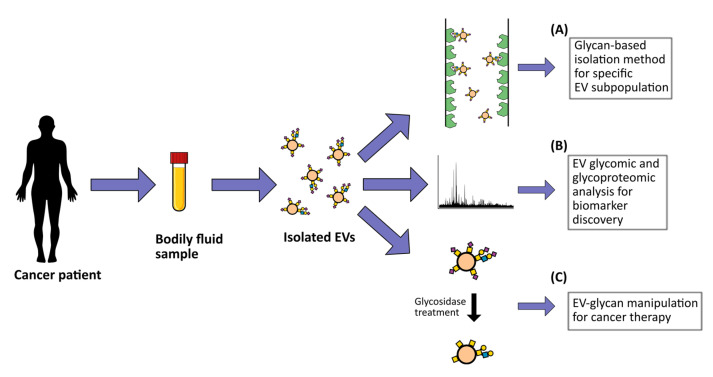
Potential clinical applications of extracellular vesicle (EV) glycosylation. These include (**A**) development of glycan-based EV detection and capture methodologies; (**B**) EV biomarker discovery for cancer diagnostic, prognostic and/or patient stratification based on EV glycomic and glycoproteomic profiling; (**C**) development of potential novel cancer therapy strategies through the manipulation of EV glycosylation surface.

Several studies have shown the possibility of using glycosylphosphatidylinositol (GPI) as an anchor to attach specific antigens to the membrane of EVs for target therapy purposes. For example, the HER-2 remained stable after its fusion with GPI and incorporation into murine breast cancer EVs, which induced strong HER-2-specific antibody responses when injected into mice [177]. In addition, fusing anti-EGFR nanobodies to the GPI anchor of neuroblastoma EVs resulted in a significantly increased capacity of these EVs to bind tumor cells that overexpress the EGFR [178]. Furthermore, the incorporation of the GPI-anchored immune-stimulatory molecule interleukin 12 (IL-12) in EVs isolated from different tumor cell lines resulted in increased in vitro T cell proliferation [179]. These studies highlight the potential of modifying the EV surface to successfully transport antigens to their destination site (Figure 2). To explore the real potential of EVs for cancer drug delivery purposes, a reliable system capable of tracking both in vitro and in vivo interactions of these natural nanoparticles is required. Interestingly, a new method of natural particle labeling based on glycan trafficking was recently reported. In this study, azido-sugars were metabolically incorporated into the cellular glycans and further packaged into the EVs, which allowed these EVs to be traceable in vivo [180].

After reaching their target destination, EVs can be incorporated by recipient cells and release their content. As discussed before, the presence of HSPGs on recipient cells has proved to act as receptors for cancer-derived EVs [9]. The structure and function of these HSPGs can be regulated by heparanase [181]. Interestingly, the use of heparanase inhibitors showed to block tumor progression by reducing exosome uptake by receptor cells [11,182,183]. Currently, there are some heparanase inhibitors, such as modified heparins or HS mimetics, whose potential use in the clinic is being tested [184,185,186,187,188]. Examples include chemically modified *N-*desulfated, *N-*acetylated and glycol-split heparin derivatives [189] and a heparanase inhibitor [190], that alone or together with lapatinib, resulted in inhibition of the tumor growth in patients with myeloma [186] or in brain metastatic breast cancer, respectively [191].

Interestingly, it was demonstrated that a specific EV glycosylation coating per se can induce a host immunogenic response. In melanoma, the modification of apoptotic EVs surface towards overexpression of high mannose type glycans, a natural ligand of DC-SIGN, increased the uptake of these EVs by monocyte-derived dendritic cells, leading to an increase in CD8+ T cell response [153]. In addition, the enzymatic removal of sialic acids and insertion of palmitoyl-Le^Y^ in glioblastoma EVs led to an enhanced EV uptake by dendritic cells in a DC-SIGN dependent manner, a receptor involved in the activation of CD8+ and CD4+ T cell responses [192]. Therefore, these studies showed that modifications of the EV glycan surface hold potential as a vaccination strategy to potentiate an anti-tumor immune response. The capacity of certain EV glycans to naturally stimulate the immune system should be further explored for the development of novel potential immune-related therapies.

A different strategy with the potential to be applied for cancer therapy involves the hemofiltration of the patient circulating exosomes using the Aethlon ADAPT™ system (adaptive dialysis-like affinity platform technology) [193]. This system aims to capture tumoral EVs through interaction with their surface proteins or glycans. The efficacy of the ADAPT™ system was evaluated in patients with end-stage renal disease. It was possible to reduce the circulating hepatitis C virus by targeting the high mannose glycans present on the viral particles [194]. Although there is no concrete data on its usage in removing EVs from the circulation of cancer patients, the ADAPT™ system is a promising strategy to capture tumor EVs based on their glycosylation profile.

The role of EVs in modulating therapeutic resistance has already been reported (as reviewed in [195]). EVs can be used by tumor cells as resistance mechanisms through the packaging and release of drugs by these vesicles [196,197,198]. Furthermore, the transfer of proteins, such as the multidrug resistance P-glycoprotein [199,200,201,202], or specific microRNAs [203,204,205] from drug-resistant cells to drug-sensitive cells can lead to the modulation of gene expression and the acquisition of resistance in recipient cells. Since glycans present on the EV surface are important mediators of the interaction and uptake of these vesicles by the recipient cells [8,9,10,11], changes in the EV glycosylation will alter the intercellular communication between resistant and sensitive cells. EVs are also capable of modulating the immune response. Through the delivery of specific cargo, such as immune-stimulatory or immune-suppressive molecules, EVs can regulate the activity of immune cells (as reviewed in [206,207]). As previously addressed in this review, glycosylation is an important modulator of the immune response, and cancer cells use specific glycan profiles to escape immunosurveillance. Thus, it is possible that tumor-derived EVs carrying these glycan signatures will also suppress the immune response.

#### 4.3.2. Cancer Biomarker Discovery

Besides their potential application for cancer therapy, EVs also represent a valuable source of circulating biomarkers (Table 1). High EV concentrations have been found in several body fluids, including blood, urine, saliva, cerebrospinal fluid, lymph, pleural effusions, semen, bronchoalveolar lavage, bile, synovial fluid, nasal secretions, breast milk, ocular effluent and ascites (as reviewed in [168]). As alterations in cell glycosylation are a common feature of cancer progression, and glycans are highly present in cancer EVs [73,208], the disclosure of cancer EV glycosylation holds a tremendous potential to identify novel reliable cancer EV biomarkers. In fact, most of the currently available cancer biomarkers are based on the detection of glycans, including the sialyl Lewis A antigen (CA19-9) and the STn (CA72-4), or glycoproteins, such as the alpha-fetoprotein (AFP), the prostate-specific antigen (PSA), mucin 16 (CA125), mucin 1 (CA15-3) and the carcinoembryonic antigen (CEA), which are used to follow both patient treatment response and tumor recurrence in several types of cancers (as reviewed in [3,4,209]).

Interestingly, the carbohydrate antigen CA125 was identified in serum-derived exosomes from patients with ovarian cancer and the detected levels were significantly higher in the exosomes when compared to the levels detected directly in the serum of these patients [210]. Yokose and his collaborators also studied the glycan profiles of serum EVs and revealed a significant increase in *O*-glycosylated EVs in pancreatic cancer patients in the early stages of the disease, even when the patient samples were negative for the CA19-9 antigen [211]. Interestingly, elevated levels of CA19-9 were detected in exosomes from pancreatic cancer patients when compared to healthy samples. The analysis of CA19-9 in exosomes proved to be more sensitive than its direct measurement from the serum, which allowed to identify CA19-9 positive exosomes in patients thought to be negative for the presence of this antigen [212]. In addition, a highly glycosylated form of the CD133 glycoprotein carrying increased levels of sialic acids was found in exosomes from pancreatic cancer patient’s ascites and was also associated with patient survival. Although further studies are needed, these results demonstrate the prognosis potential of CD133-specific glycosylation in pancreatic cancer [213]. In addition to glycoproteins, the glycosphingolipids abundantly present on the surface of prostate cancer EVs have also been reported as promising biomarkers for this type of cancer [214].

The overexpression or de novo synthesis of particular glycans or glycoconjugates during cancer progression holds the potential to differentiate tumor EVs from benign EVs. Indeed, the proteoglycan glypican-1 (GPC1) and the tumor antigen chondroitin sulfate proteoglycan 4 (CSPG4) were detected in tumor exosomes from heterogeneous samples of pancreatic cancer [155] or melanoma [156], respectively. In both cases, these glycoproteins were able to differentiate tumor-derived EVs from non-malignant particles. In a recent study, the proteoglycan versican (VCAN) and the glycoprotein tenascin C (TNC) also proved to be able to distinguish tumor from non-tumor tissues with high sensitivity and specificity, pointing to their use as cancer EVs markers [215]. In the same study, the galactoside-binding soluble 3 binding protein (LGALS3BP) was identified in most of the EV samples [215], which is in line with the previous reports of the presence of LGALS3BP in uveal melanoma [123] and ovarian cancer EVs [72,111]. Interestingly, the LGALS3BP protein was also found to be strongly enriched in the recently discovered cancer exomere particles [91]. Moreover, the glycoprotein basigin (CD147 or EMMPRIN) and the proteoglycan biglycan (BGN) were found enriched in pancreatic tumor EVs when compared to EVs secreted by non-tumor adjacent tissues [215]. In accordance, highly glycosylated variants of EMMPRIN were predominantly detected on cancer patient-derived microvesicles and were positively correlated with poor survival in several types of cancer [216].

In addition, the *O*-GlcNAc glycosylation has also been found elevated in breast [154] and colorectal cancer EVs [88], when compared to normal conditions. In particular, the *O*-GlcNAc modification of the transitional endoplasmic reticulum ATPase (TER ATPase) and RuVB-like1 proteins was identified in colorectal metastatic EVs [88]. Elevated levels of *O*-GlcNAc were also detected in TER ATPase as well as in 70 kDa heat-shock protein (HSP70) proteins present in breast cancer EVs, which may act to protect cytosolic and nuclear proteins against degradation [154]. The elevated presence of this type of glycosylation modification conjugated with specific proteins identified in tumoral EVs when compared to normal conditions suggests its potential use as a biomarker for both breast cancer and metastatic colorectal cancer.

Despite the high potential of EV glycosylation in the discovery of novel cancer biomarkers, studies addressing the glycan profile of blood circulating EVs are still quite scarce. Nevertheless, the *N*-glycome of exosomes from hepatocellular carcinoma patient samples was characterized using a reverse capture strategy, and the majority of the *N*-glycans found in EVs from patients with HCC were modified with sialic acids or fucoses, in contrast to the *N*-glycans identified in EV from healthy samples [151]. Walker et al. also reported significant differences between the glycan profiles identified directly in the plasma or the plasma-derived EVs from the same individuals [217]. Interestingly, a new integrated analytical platform, termed the integrated magnetic analysis of glycans in extracellular vesicles (iMAGE), was developed to directly analyze the EV glycosylation profile in biological samples. This platform aims to facilitate the EV glycome analysis taking advantage of the magnetic nanotechnologies [218]. The effectiveness of this strategy was evaluated by spiking kidney and brain cancer-EVs into urine and serum EV-depleted samples, respectively, and analyzing the glycan signatures. This strategy proved to be efficient in detecting EVs. Subsequently, when analyzing the glycan profile of ascites samples from patients with gastric and colorectal cancer, it was possible to distinguish patients based on their prognosis only by the glycans present at the EV surface. The distinction of these patients was possible by the increased signal of different lectins associated with a poor prognosis, including the Jacalin, ConA, RCA120, PHA-E, STA, LEL, WGA, DSL and LCA lectins. Although further studies are needed to prove the iMAGE platform’s robustness, this new method of profiling glycans may prove to be very useful in the search for novel biomarkers in cancer research [218]. Although several techniques can be used to analyze glycans, their study faces several technical challenges. The most commonly used methods only provide relative and not absolute quantification of the glycans present in a sample and are often based on a targeted search for specific patterns of cancer-associated glycans [219]. In addition, the variability of the results obtained when the same samples are analyzed in different laboratories, in which different methods were used, demonstrates the difficulty in choosing the best methodology, and the need for reference standards that support that choice [220]. Nevertheless, the presence of different glycosylation profiles under normal and cancer conditions highlights the potential of studying EV-specific glycosylation for the identification of novel cancer circulating biomarkers. Indeed, the studies addressing EV glycosylation denote a high potential of EV glycans to distinguish from normal vs. tumoral EVs. Therefore, we believe further in-depth studies of EV glycosylation will bring several benefits for the future of cancer patients.

## 5. Discussion

Glycosylation is a post-translational modification that plays a variety of roles in both physiological and pathological contexts [221]. Interestingly, some of the aberrant glycosylation signatures acquired during cancer progression have been identified in cancer EVs [86]. These discoveries have boosted a worldwide effort to characterize the glycome of tumor-derived EVs and use this knowledge to develop innovative methods for both EV detection and isolation based on specific glycosylation signatures. Challenges stemming from the structural complexity of glycans and the low yield of current EV isolation methods have hindered our ability to fully elucidate and understand their biological role during disease progression. Nevertheless, different studies already gave insights into the biological roles of altered glycosylation during EV uptake, biodistribution and protein cargo sorting. Indeed, these biological processes were reported to be relevant in cancer progression [169]. Furthermore, EVs constitute an accessible source of glycans and specific glycans/glycoproteins signatures with the potential to predict their origin, and therefore to potentially distinguish EVs released from healthy or cancer tissues or even to identify the type of cancer of origin. Thus, studies characterizing EV glycosylation profiles of healthy individuals and cancer patients resorting to high-resolution methods are a need to identify potential novel cancer biomarkers. As an important remark, the standardization of current EV isolation/characterization protocols across the world remains a milestone to uniformize and ensure the reproducibility of studies conducted in the field, especially those aiming to be translated into the clinical setting [222].

There is still a large number of questions to be answered regarding the functional role of EV glycosylation in cancer as well as challenges on how to address these questions. Future studies aiming to provide these answers will allow us to discover potential EV glycan-based biomarkers for cancer diagnosis/prognosis/patient stratification and develop innovative therapeutic strategies that can be applied for cancer treatment.

## Figures and Tables

**Table 1:** List of the main structures of glycans identified in different cancer extracellular vesicles (EVs) samples and their relevance and potential impact in the EV field.

Glycan Structure	Cancer Type	Sample	Relevance/Potential Impact	References
**GalNAcα-Thr/Ser (Tn antigen)**	Cervical	Cell lines	EV biomarker potential	[87]
**Neu5Acα(2,6)-GalNAcα-Thr/Ser (STn antigen)**	Gastric, cervical	Cell lines	EV biomarker potential	[86,87]
**Gal-β(1,3)-GalNAcα-Thr/Ser (T antigen)**	Ovarian, cervical	Cell lines	EV detection EV biomarker potential	[72,87]
**Sialylation (α(2,6)- or α(2,3)-linked)**	Hepatic, melanoma, cervical, pancreatic, ovarian, colorectal, glioma, breast, gastric	Cell lines, human serum, healthy individuals’ urine	EV detection and capture EV biomarker potential EV uptake EV biodistribution	[5,6,8,70,71,72,84,86,87,89,91,111,122,123,131,144,148,150,151,152]
**Core fucosylated *N*-glycans**	Hepatocellular, breast, glioma, gastric melanoma	Cell lines, human serum	EV detection EV biomarker potential	[71,84,86,87,91,151]
**Terminal fucosylation**	Melanoma, breast	Cell lines	EV detection	[5,91]
**Complex *N*-glycans**	Melanoma, colorectal, hepatocellular	Cell lines, human serum, healthy individuals’ urine	EV detection EV biomarker potential	[6,70,84,89,151]
**Branched *N*-glycans**	Pancreatic, melanoma, breast, ovarian, gastric	Cell lines	EV detection EV biomarker potential	[5,84,86,91,111,123,131]
**Bisected *N*-glycans**	Melanoma, pancreatic, ovarian, gastric	Cell lines, healthy individuals’ urine	EV detection EV biomarker potential	[5,70,72,86,91,123]
**High mannose *N*-glycans**	Melanoma, glioblastoma, lung, colorectal, ovarian, hepatocellular	Cell lines, human serum, healthy individuals’ urine	EV detection and capture EV therapy potential EV biomarker potential EV uptake	[6,70,72,89,111,123,144,151,153]
**Polylactosamine**	Colorectal, melanoma	Cell lines	EV detection	[6]
***O*-GlcNAc**	Breast, colorectal	Cell lines	EV biomarker potential	[88,154]
**GAGs**	Endometrial, ovarian, breast	Cell lines	EV capture	[149]
**Proteoglycans**	Pancreatic, melanoma, breast	Cell lines, human and mice serum, human plasma	EV capture EV biomarker potential	[155,156]

EV, extracellular vesicles; GalNAc, *N*-Acetylgalactosamine; Thr, threonine; Ser, serine; Neu5Ac, *N*-Acetylneuraminic acid; STn, sialyl-Tn; Gal, galactose; *O*-GlcNAc, *O*-GlcNAcylation; GAGs, glycosaminoglycans.

## Data Availability

No new data were created or analyzed in this study. Data sharing is not applicable to this article.

## Abbreviations

AF4	asymmetric-flow field-flow fractionation
AFP	alpha-fetoprotein
BGN	biglycan
CEA	carcinoembryonic antigen
CSPG4	tumor antigen chondroitin sulfate proteoglycan 4
DC-SIGN	dendritic cell-specific intercellular adhesion molecule-3-grabbing non-integrin
ECM	extracellular matrix
EFF	evanescent-field fluorescence-assisted
EGFR	epidermal growth factor receptor
EMMPRIN	extracellular matrix metalloproteinase inducer
ER	endoplasmic reticulum
ESCRT	endosomal sorting complex required for transport
EVs	extracellular vesicles
FasL	fas ligand
GA	golgi apparatus
GAGs	glycosaminoglycans
GPC1	glypican-1
GPI	glycosylphosphatidylinositol
HCC	hepatocellular carcinoma
HER2	human epidermal growth factor receptor 2
HGF	hepatocyte growth factor
HS	heparan Sulfate
HSPGs	heparan sulfate proteoglycans
IL-12	interleukin 12
iMAGE	integrated magnetic analysis of glycans in extracellular vesicles
LC/MS-MS	liquid chromatography/tandem mass spectrometry
LCN2	lipocalin 2
LGALS3BP	galactoside-binding soluble 3 binding protein
MALDI TOF	matrix-assisted laser desorption ionization time-of-flight
MAL-I	*Maackia amurensis* lectin I
MAPK	mitogen-activated protein kinase
MVBs	multivesicular bodies
NK	Natural Killer
NKG2D	Natural Killer group 2D
NP-TRFIA	nanoparticle-based time-resolved fluorescence immunoassay
PD-L1	programmed death-ligand 1
PNA	peanut agglutinin
PSA	prostate-specific antigen
RTK	receptor tyrosine kinase
SC	simple-cell
Siglecs	sialic acid binding immunoglobulin-type lectins
STn	Sialyl-Tn
TER ATPase	transitional endoplasmic reticulum ATPase
TGF-β	transforming growth factor-β
THP	Tamm–Horsfall protein
TMZ	alkylating agent temozolomide
TRAIL	TNF-related apoptosis-inducing ligand
VEGF	vascular endothelial growth factor
VEGFR	vascular endothelial growth factor receptor
